# Anonymous Communications

**Published:** 1842-12

**Authors:** 


*Anonymous Communications.*—As some craven-hearted writers, who seek to assail personal and professional character under the mask of anonymous communications, may feel some astonishment at never seeing their productions either published or noticed, it may be well for us to mention an invariable rule to which we have bound ourselves in all cases; that is, on the reception of such anonymous writing, affecting character and reputation, to read just enough of the commencing paragraphs to ascertain the fact of its so relating to the conduct or general character of an individual whose name is given, then turn to the conclusion and see if a responsible signature can be found; and, if not, to commit the manuscript immediately to the flames, not suffering a single idea or suspicion it may breathe to enter into and obtain a lodgment in the mind any more than the poisonous breath of the basilic would be entertained and inhaled into the system. A widely extended knowledge of our course may save some paper, and prevent the shameful insult to our understanding and manliness of character, of conceiving it possible to originate a thought of our heart, or an act of our conduct, by such hidden and assassin-like means.

Any person who has a laudable object to promote cannot be afraid of being known, either as the author of important information, or the accuser of some one's character; and any one who communicates the scandal, without daring to attach his name, brands himself as a slanderer and would-be-assassin in the dark, and must be so considered by every one of noble and generous sentiments. The time has passed when the propagater of scandal can thrive in secret; he must come out boldly, meet the consequences, establish his assertions, or consent to be looked upon as a noisome thing, fit only to be despised and trodden under foot.

The foregoing remarks have been elicited by a communication addressed to us, mailed at New York. We believe we know the author, and have preserved a line of the letter for the purpose of ascertaining if our suspicions are correct, and should they prove to be so, we may hold him up to the scorn his dastardly and pusillanimous conduct so justly merits.

We were informed some months since that a gentleman of high standing in the dental profession, and one for whom we entertain a high regard, but not a member of the American Society of Dental Surgeons, received a most impudent and insulting anonymous letter, purporting to have emanated from a member of said society. We should be exceedingly sorry to think that any one had been admitted to membership in this society capable of so mean an act, and if we could ascertain who the individual was that had done it, if he were a member of the society, we would vote for his immediate expulsion.

